# Validation of the German version of the Dimensional Apathy Scale (G‐DAS): Application in amyotrophic lateral sclerosis

**DOI:** 10.1111/jnp.70061

**Published:** 2026-06-25

**Authors:** Judith Wesenberg, Paula Matthies, Kati Schwiecker, Verena Bittner, Samra Hamzic, Stefan Vielhaber, Ratko Radakovic

**Affiliations:** ^1^ Institute of Cognitive Neurology and Dementia Research Otto‐von‐Guericke University Magdeburg Magdeburg Germany; ^2^ Center for Behavioral Brain Sciences Otto‐von‐Guericke University Magdeburg Magdeburg Germany; ^3^ Department of Neurology Otto‐von‐Guericke University Magdeburg Magdeburg Germany; ^4^ Department of Neurology Justus‐Liebig‐Universität Giessen Giessen Germany; ^5^ Swiss University of Speech and Language Sciences (hlo) St. Gallen Switzerland; ^6^ Faculty of Medicine and Health Sciences University of East Anglia Norwich UK; ^7^ Cambridgeshire and Peterborough NHS Foundation Trust Cambridge UK; ^8^ Alzheimer Scotland Dementia Research Centre Edinburgh UK; ^9^ Euan MacDonald Centre for Motor Neuron Disease Research University of Edinburgh Edinburgh UK

**Keywords:** ALS, apathy, Dimensional Apathy Scale, validation

## Abstract

Apathy is a common behavioural impairment in neurodegenerative conditions and is conceptualized within the Dimensional Apathy Framework as comprising Executive, Emotional and Initiation subtypes. The Dimensional Apathy Scale (DAS) is widely used to assess these domains, yet no validated German version has been available. This study aimed to translate and validate the German DAS (G‐DAS) in control participants (HC) and to characterize apathy profiles in German‐speaking people with amyotrophic lateral sclerosis (pwALS). Seventy‐seven HC and 32 pwALS completed self‐rated and caregiver‐rated measures of apathy, depression, disinhibition and executive dysfunction. The G‐DAS was translated using a multi‐round back‐translation procedure. Psychometric validation was undertaken in the HC cohort. A subsample of HC matched to pwALS on age and sex was used for between‐group comparisons and for deriving exploratory reference thresholds. The G‐DAS demonstrated good to high internal consistency across subscales (*α* = .76–.85) and total scores (self‐rated: *α* = .88; caregiver‐rated: *α* = .86). Convergent validity was supported by significant correlations with the Apathy Evaluation Scale and Frontal Systems Behavior subscales, particularly for the Initiation and Executive subscales. Divergent validity was evidenced by the absence of associations with anxiety and depression. PwALS showed significantly higher Executive and Initiation apathy compared with matched HC, whereas Emotional apathy did not differ. Exploratory threshold scores derived from matched HC indicated that up to 47% of pwALS exhibited clinically elevated Initiation apathy. The G‐DAS is a reliable and valid German‐language measure of multidimensional apathy. It effectively captures the characteristic Executive and Initiation apathy profile in ALS, supporting its clinical and research utility.

## INTRODUCTION

Apathy is a multidimensional construct characterized by reduced goal‐directed behaviour, cognition and emotional engagement (Marin, 1996), and is common in neurodegenerative conditions. According to the Dimensional Apathy Framework (DAF) (Radakovic & Abrahams, 2018), apathy consists of three subtypes: Executive (lack of motivation for planning, organization or attention), Emotional (indifference, affective or emotional neutrality, flatness or blunting) and Initiation (lack of motivation for self‐generated thoughts and/or behaviours). This framework is derived from converging evidence across methodologies (Radakovic & Abrahams, 2018).

The Dimensional Apathy Scale (DAS) was developed to assess these subtypes and has been validated across various psychiatric, neurological and neurodegenerative conditions, controlling for physical disability (Radakovic et al., 2016). Amyotrophic lateral sclerosis (ALS), a progressive neurodegenerative disease with frontotemporal involvement, represents a particularly relevant clinical model of apathy due to its prevalence and functional impact, with Initiation apathy being especially prominent (Radakovic et al., 2016).

The DAS has been translated and validated in several languages (M'Barek et al., 2020), for example Italian (Santangelo, Raimo, et al., 2017a). However, no validated German version is currently available. Therefore, the present study aimed to translate and validate the German version of the DAS (G‐DAS) and explore apathy profiles among German‐speaking people with ALS (pwALS).

## METHODS

### Participants

Seventy‐seven healthy controls (HCs) and 32 pwALS were recruited from the Universities of Magdeburg and Gießen (2021–2024). pwALS had clinically confirmed ALS (El Escorial criteria) diagnosed by experienced neurologists. Clinical characterization included ALSFRS‐R and disease duration. HCs were recruited via advertisement and chain‐referral sampling. Caregivers provided proxy ratings but were not included as HC participants. Eligibility criteria were age 35–80 years and no history of neurological or psychiatric disease.

### Procedure

Demographic and clinical data were collected for all participants. Measures were completed in clinic or at home. No a priori power analysis was conducted due to the exploratory design and limited ALS sample; the sample size is comparable to previous DAS validations.

### Measures

Secondary measures assessed convergent (apathy) and divergent (mood, disinhibition) validity using established instruments.

#### Apathy

The G‐DAS (self‐rated and caregiver‐rated) is a 24‐item multidimensional apathy measure assessing Executive, Emotional and Initiation apathy (8 items each). Items are rated on a 4‐point Likert scale (0–3) and total scores range from 0 (no apathy) to 24 (most apathy). The translation of the original DAS (Radakovic & Abrahams, 2014; Radakovic et al., 2016) was conducted through a 4‐round iterative back‐translation process involving professional German‐English bilinguals and translators to ensure both linguistic accuracy and cultural equivalence (Figure S1). The G‐DAS (self‐rated and caregiver‐rated) and scoring sheet can be found in the Supporting Information, as well as a detailed flow chart of the translation process.

The Apathy Evaluation Scale (AES‐D, self‐rated and caregiver‐rated) is an 18‐item measure (Lueken et al., 2006) using a 4‐point Likert scale, with scores ranging from 18 (no apathy) to 72 (most apathy).

#### Depression

The Hospital Anxiety and Depression Scale (HADS) is a 14‐item self‐rated measure for assessing anxiety and depression. The depression subscale ranges from 0 (no depression) to 21 (most depression), and the anxiety subscale ranges from 0 (no anxiety) to 21 (most anxiety). Items are rated on a 4‐point Likert scale (0–3).

#### Frontal systems behaviours

The Frontal Systems Behavior Scale (FrSBe) (46‐items, self‐rated and caregiver‐rated) (Grace & Malloy, 2001) assesses apathy, disinhibition and executive dysfunction. Items are rated on a 5‐point Likert scale. In pwALS, both current and pre‐illness ratings were obtained; analyses used current ratings for comparability with HC.

#### Functional disability

The ALS Functional Rating Scale‐Revised (ALSFRS‐R) is a 12‐item measure (Cedarbaum et al., 1999) that assesses the extent of physical disability of pwALS. Total scores range from 0 (worst functioning) to 48 (normal functioning). The ALSFRS‐R was administered by an experienced senior neurologist.

### Statistical analysis

R statistical software (version 4.5.1) was used for all analyses. Data distribution was assessed using visual inspection and Shapiro–Wilk tests (Table S1) and informed the use of non‐parametric analyses. All analyses were corrected for multiple comparisons (Bonferroni correction), with the significance threshold set at *p* < 0.05.

The G‐DAS validation was performed exclusively in the HC group. The G‐DAS internal consistency reliability (self‐rated and caregiver‐rated versions) was assessed using Cronbach's alpha, with values ≥ 0.70 considered acceptable. Spearman's rho correlation was used to determine self‐rated and caregiver‐rated G‐DAS convergent (AES‐D, FrSBe‐apathy subscale) and divergent (HADS, FrSBe‐disinhibition subscale) validity.

For comparisons between pwALS and HC, a subsample of HC participants (HC‐matched) was derived from the full HC cohort using age‐ and sex‐based matching implemented via the MatchIt package in R. No additional inclusion or exclusion criteria were applied beyond those used for the original HC sample. Group comparison between pwALS and HC‐matched was done using Wilcoxon rank‐sum tests. The HC‐matched G‐DAS scores were used to derive exploratory reference thresholds (mean + 2 standard deviations) to indicate elevated apathy levels within the sample.

## RESULTS

### Demographics

Table 1 shows demographic, clinical and measure (self‐rated and caregiver‐rated) comparisons for the HC validation cohort, the HC‐matched group and pwALS.

**TABLE 1:** Demographic, clinical and measure comparison between HC, HC‐matched and pwALS.

	HC	*N*	HC‐matched	*N*	pwALS	*N*	*W*	HC‐matched < pwALS *p*
Age (median, range)	64.5 (35–87)	74	67.0 (45–87)	32	67.5 (47–78)	32	486.5	0.737
Sex, N (f/m)	39/35	74	12/20	32	12/20	32		
ALSFRS‐R (median, range)	na		na		40.0 (21–46)	32		
Disease duration [months] (median, range)	na		na		15.1 (3.3–215)	32		
ECAS (median, range)	115 (101–128)	35	118 (108–128)	16	102 (36–126)	24	**324**	**< .001**
AES self (median, range)	24.0 (10–56)	69	23.0 (18–49)	32	29.0 (19–54)	23	**169**	.**001**
AES caregiver (median, range)	24.0 (18–55)	62	23.0 (3–19)	32	33.0 (15–54)	25	**144**	**< .001**
FrSBe self‐total (median, range)	79.0 (48–134)	71	76.5 (48–117)	32	82.0 (57–119)	23	314	0.361
FrSBe self‐apathy (median, range)	23.0 (14–48)		21.5 (14–38)		31.0 (11–53)		**223**	**0.014**
FrSBe self‐disinhibition (median, range)	26.0 (15–40)		26.0 (15–37)		22.0 (15–35)		**490**	**0.037**
FrSBe self‐executive dysfunction (median, range)	29.0 (17–57)		28.0 (17–48)		31.0 (20–51)		315.5	0.370
FrSBe caregiver total (median, range)	75.0 (50–113)	59	74.5 (51–111)	32	87.0 (58–142)	26	**200**	**0.005**
FrSBe caregiver apathy (median, range)	20.0 (13–32)		20.0 (14–32)		29.0 (22–52)		**82.5**	**< .001**
FrSBe caregiver disinhibition (median, range)	24.0 (15–42)		23.5 (15–34)		22.0 (15–36)		424	0.302
FrSBe caregiver executive dysfunction (median, range)	29.0 (17–52)		29.0 (17–52)		35.5 (18–64)		**245**	**0.040**
HADS total (median, range)	8.0 (0–27)	74	7.5 (0–16)	32	14.0 (1–35)	28	**232.5**	.**001**
HADS anxiety (median, range)	4.0 (0–15)		4.0 (0–9)		7.5 (0–17)		**278**	**0.012**
HADS depression (median, range)	4.0 (0–12)		3.0 (0–9)		7.0 (1–18)		**204.5**	**< .001**

*Note:* Bold values indicate significance levels below *p* < .05.

### Psychometrics

#### Reliability

The G‐DAS demonstrated high overall internal consistency reliability in both the self‐rated (*α* = .88) and caregiver‐rated (*α* = .86) versions. All three G‐DAS subscales showed acceptable to high reliability coefficients: Executive (self‐rated: *α* = .85, caregiver‐rated: *α* = .81), Emotional (self‐rated: *α* = .77, caregiver‐rated: *α* = .77) and Initiation (self‐rated: *α* = .80, caregiver‐rated: *α* = .76). Item‐subscale correlations are displayed in Table S1.

#### Convergent and divergent validity

**FIGURE 1:**
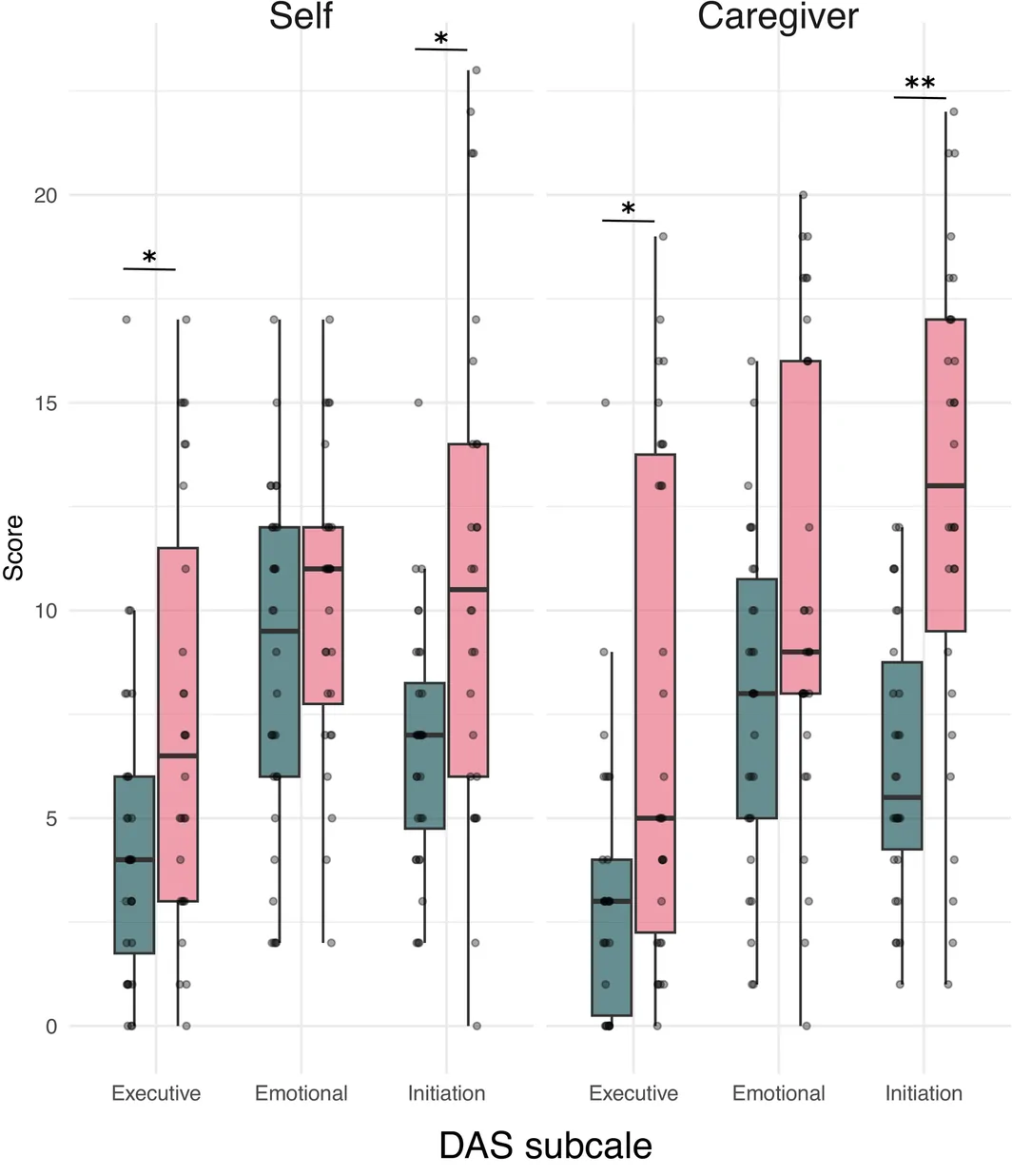
Comparison between pwALS and HC‐matched subscale sums in the G‐DAS self‐rated (left) and caregiver‐rated (right) versions. *p < 0.05; **p < .001.

The G‐DAS total score for both the self‐rated and caregiver‐rated versions showed significant correlations with the AES‐D (self‐rated: *r* = .66, *p* < .001; caregiver‐rated: *r* = .72, *p* < .001), the FrSBe‐total score (self‐rated: *r* = .66, *p* < .001; caregiver‐rated: *r* = .66, *p* < .001), the FrSBe‐apathy subscale (self‐rated: *r* = .61, *p* < .001; caregiver‐rated: *r* = .52, *p* < 001) and executive dysfunction (self‐rated: *r* = .67, *p* < .001; caregiver‐rated: *r* = .61, *p* < .001). Additionally, the G‐DAS total score was correlated with the FrSBe‐disinhibition subscale, but only in the caregiver‐rated version (*r* = .54, *p* < 0.001, Figure 1).

The G‐DAS Executive subscale was significantly correlated with the AES‐D (self‐rated: *r* = .61, *p* < .001; caregiver‐rated: *r* = .51, *p* < .001), FrSBe‐total score (self‐rated: *r* = .74, *p* < .001; caregiver‐rated: *r* = .62, *p* < .001), FrSBe‐disinhibition (self‐rated: *r* = .68, *p* < .001; caregiver‐rated: *r* = .64, *p* < .001) and FrSBe‐executive subscale (self‐rated: *r* = .64, *p* < .001; caregiver‐rated: *r* = .56, *p* < .001). In the self‐rated version, the G‐DAS Executive subscale was also correlated with the FrSBe‐apathy subscale (*r* = 0.68, *p* < 0.001).

The G‐DAS Emotional subscale was only correlated with the FrSBe‐executive subscale in the self‐version (*r* = 0.46, *p* = 0.003) and with the AES‐D in the caregiver‐rated version (*r* = 0.44, *p* = .025, Figure 1).

The G‐DAS Initiation subscale was correlated with the AES‐D (self‐rated: *r* = .67, *p* < .001; caregiver‐rated: *r* = .67, *p* < .001), FrSBe‐total (self‐rated: *r* = .41, *p* = .028; caregiver‐rated: *r* = .46, *p* = .014) and FrSBe‐apathy subscale (self‐rated: *r* = .46, *p* = .003; caregiver‐rated: *r* = .44, *p* = .035, Figure 1).

Neither the total G‐DAS nor the subscales were correlated with the HADS‐depression, HADS‐anxiety or HADS‐total scores. Additionally, the FrSBe‐disinhibition subscale was not significantly correlated with the G‐DAS Initiation and Emotional apathy subscales, for both self‐rated and caregiver‐rated versions (Figure 1). Item‐subscale correlations are available in Table S2.

### Apathy subtypes in pwALS

**FIGURE 2:**
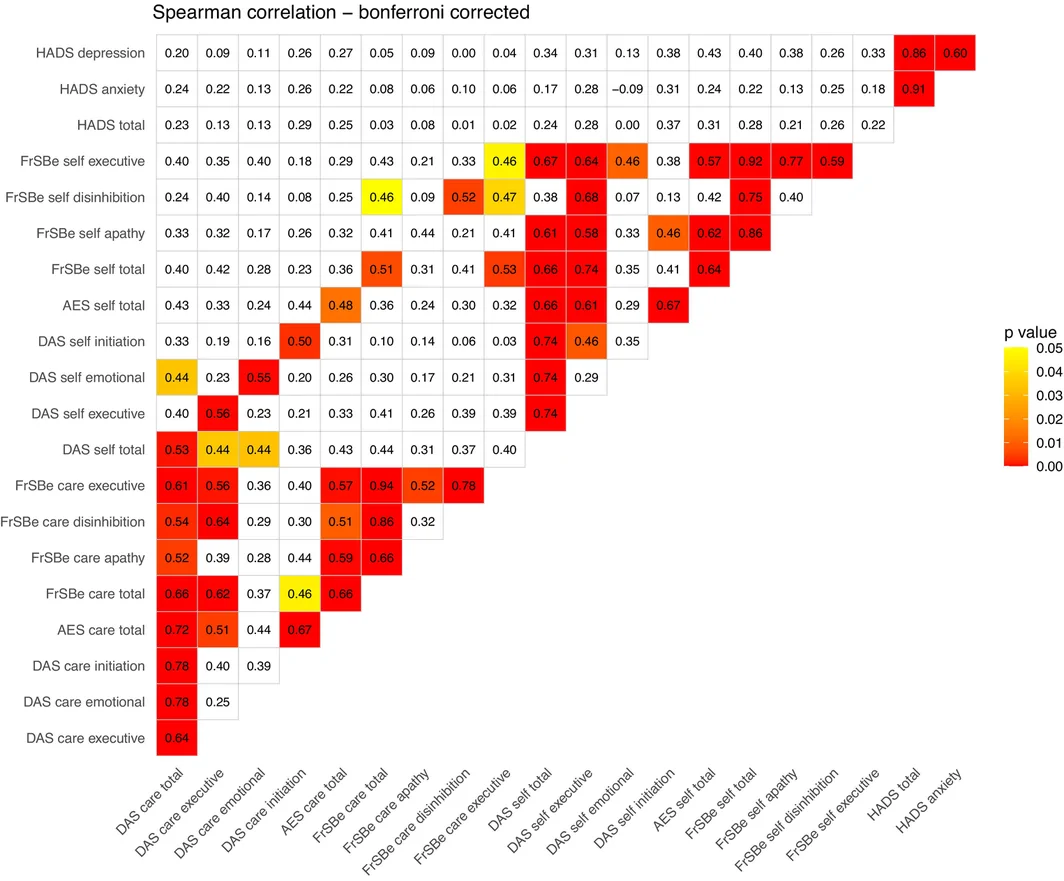
Spearman correlations between all conducted questionnaires in the self‐rated (left) and caregiver‐rated (right) versions. The colour bar shows the *p*‐value only for significant correlations following Bonferroni correction (*p* < 0.05).

Figure 2 shows that pwALS exhibited significantly higher levels of Executive (self‐rated: *p* = 0.018, caregiver‐rated: *p* = 0.003) and Initiation (self‐rated: *p* = 0.003, caregiver‐rated: *p* < .001) apathy scores compared to the HC‐matched group, with no difference in Emotional apathy (self‐rated: *p* = 0.283, caregiver‐rated: *p* = 0.057). See Table S3 for raw scores.

HC‐matched derived G‐DAS reference thresholds were: Executive apathy ≥ 12 for self‐rated and ≥ 11 for caregiver‐rated, Emotional apathy ≥ 15 for both, self‐rated and caregiver‐rated, and Initiation apathy ≥ 12 for both, self‐rated and caregiver‐rated, with the G‐DAS Total ≥ 33 for self‐rated and ≥ 31 for caregiver‐rated. 34.4% and 21.9% of pwALS displayed Executive apathy (caregiver‐rated and self‐rated, respectively). Further, 31.3% and 3.2% of pwALS displayed Emotional apathy (caregiver‐rated and self‐rated, respectively). Finally, 46.9% and 37.5% of pwALS displayed Initiation apathy (caregiver‐rated and self‐rated, respectively).

## DISCUSSION

The present study translated and validated the German language DAS (G‐DAS), both self‐rated and caregiver‐rated versions, demonstrating robust reliability and validity. Its ability to distinguish specific apathy subtype profiles, particularly Executive and Initiation apathy in pwALS, further supports its suitability for research and clinical use.

The G‐DAS subscales showed good reliability (internal consistency) comparable to previous validation studies (Santangelo, Raimo, et al., 2017a). Good convergent validity was found for the G‐DAS with strong correlations with the AES‐D and FrSBe, indicating that the G‐DAS captures motivational and executive aspects of apathy. The associations were particularly pronounced for the Initiation and Executive subscales, consistent with previous findings (Radakovic et al., 2016). The weaker, variable correlations with the Emotional subscale indicate that this domain reflects a distinct component of apathy, with previous research proposing additional subconstructs (M'Barek et al., 2020) that appear less strongly linked to cognitive or behavioural disinhibition. However, divergent validity was supported by the absence of associations between the G‐DAS (particularly Emotional Apathy) and anxiety or depression symptoms, suggesting that apathy may be distinguishable from affective difficulties. Further, the lack of correlation between the Emotional or Initiation subscales and the FrSBe‐disinhibition domain supports the G‐DAS capturing of goal‐directed motivational deficits rather than dysregulation difficulties. This finding suggests that the G‐DAS was psychometrically robust, successfully preserving the conceptual coherence of the original scale (Radakovic & Abrahams, 2014) and capturing the underlying DAF concept (Radakovic & Abrahams, 2018).

The observed ALS apathy profile was characterized by heightened Initiation and Executive subtypes compared with matched controls, consistent with earlier work (Santangelo, Raimo, et al., 2017a; Santangelo, Siciliano, et al., 2017b) and highlights the cross‐linguistic robustness of the DAF (Radakovic et al., 2016). The application of sample‐based, demographically matched G‐DAS reference thresholds provides additional descriptive context, echoing patterns reported in prior research (Radakovic et al., 2016); however, the derived G‐DAS reference thresholds were generally lower than those reported in the original and other language versions of the DAS (Radakovic et al., 2016), particularly for the Executive and Initiation subscales, while Emotional apathy thresholds showed closer agreement across studies (M'Barek et al., 2020; Radakovic et al., 2016; Santangelo, Raimo, et al., 2017a). These differences likely reflect methodological factors, including variations in sample characteristics and language adaptation. Accordingly, these thresholds should be interpreted as sample‐specific, exploratory reference values rather than directly comparable clinical cut‐offs.

Further, the observed variable correlations of apathy subtypes with frontal systems behaviours (i.e., executive dysfunction and disinhibition) are parallel with apathy‐cognitive functioning overlap in ALS (Radakovic & Abrahams, 2018) but may warrant further exploration. The observed apathy profiles should be interpreted in light of the heterogeneous neuropsychological phenotypes within the ALS cohort.

This study is not without limitations. While the pwALS sample size was modest, future studies should examine the G‐DAS in larger German‐speaking cohorts across neurodegenerative conditions to allow more in‐depth profiling of apathy and its correlates. In addition, no structural analysis (e.g., exploratory or confirmatory factor analysis) was conducted; although the factor structure of the original DAS is well established, future research should confirm the dimensional structure of the German version. Furthermore, other relevant psychometric properties, such as test–retest reliability, were not assessed and should be examined in future studies. Finally, the relatively small and older‐skewed HC sample may have influenced the derived thresholds, although age showed no meaningful association with G‐DAS scores.

In conclusion, the G‐DAS is a reliable and valid instrument for assessing multidimensional apathy, and its application in pwALS demonstrates its ability to capture increased Initiation and Executive apathy. These findings support its utility in ALS and provide a foundation for extending the G‐DAS to other clinical populations to further advance profiling of apathy subtypes.

## AUTHOR CONTRIBUTIONS

**Judith Wesenberg:** Conceptualization; project administration; formal analysis; funding acquisition; investigation; methodology; writing – original draft; writing – review and editing. **Paula Matthies:** Investigation; project administration. **Kati Schwiecker:** Investigation. **Verena Bittner:** Investigation; writing – review and editing. **Samra Hamzic:** Conceptualization; methodology; resources; investigation; funding acquisition; writing – review and editing. **Stefan Vielhaber:** Writing – review and editing; investigation; resources. **Ratko Radakovic:** Conceptualization; methodology; formal analysis; writing – original draft; writing – review and editing.

## FUNDING INFORMATION

JW has been funded by the federal state of Saxony‐Anhalt and the European Regional Development Fund (ERDF) in the Center for Behavioral Brain Sciences (CBBS, ZS/2016/04/78113).

## CONFLICT OF INTEREST STATEMENT

The authors declare no conflict of interest.

## ETHICS STATEMENT

The study was approved by the local ethics committee of Otto‐von‐Guericke University (Ref. No. 136/20).

## CONSENT

All participants gave written informed consent.

## Supporting information


**Table S1.** Shapiro–Wilk test results for individual variables.
**Table S2.** Item‐subscale correlations.
**Table S3.** Median, IQR and range for G‐DAS subscales separated for self‐rated and caregiver‐rated versions in HC‐matched and pwALS.
**Figure S1.** Detailed flow chart of the translational process. During this process, items were reviewed for linguistic clarity and cultural relevance. No substantial cultural adaptations were required as the items were considered applicable in the German context; only minor linguistic refinements were made to improve clarity and natural phrasing (i.e., Item 15 was phrased positively rather than the original DAS version).

## Data Availability

The data supporting the findings of this study contain sensitive information and are therefore not publicly available. Anonymized data may be shared with qualified researchers upon reasonable request to the corresponding author, subject to institutional and ethical approval.
