# Supplementary material for: Validation of the German version of the Dimensional Apathy Scale (G‐DAS): Application in amyotrophic lateral sclerosis
Source: J Neuropsychol. 2026 Jun 25;20(3):702–12. doi: 10.1111/jnp.70061 (PMC13552254; doi:10.1111/jnp.70061)
Supplement: Supplementary file 1 [file JNP-20-702-s001.docx]

Supplementary Table 1: Shapiro-Wilk test results for individual variables

| Variable | Group | N | W | p value |
| --- | --- | --- | --- | --- |
| Age | pwALS | 32 | 0.942 | 0.0835 |
| DAS care total | pwALS | 30 | 0.967 | 0.4533 |
| DAS care Executive | pwALS | 30 | 0.884 | **0.0035** |
| DAS care Emotional | pwALS | 30 | 0.928 | **0.0426** |
| DAS care Initiation | pwALS | 30 | 0.958 | 0.281 |
| DAS self total | pwALS | 28 | 0.916 | **0.027** |
| DAS self Executive | pwALS | 28 | 0.921 | **0.0376** |
| DAS self Emotional | pwALS | 28 | 0.978 | 0.7905 |
| DAS self Initiation | pwALS | 28 | 0.96 | 0.3426 |
| HADS-D total | pwALS | 28 | 0.964 | 0.4289 |
| HADS-D Anxiety | pwALS | 28 | 0.965 | 0.4542 |
| HADS-D Depression | pwALS | 28 | 0.926 | **0.0486** |
| AES self | pwALS | 23 | 0.913 | **0.0466** |
| AES care | pwALS | 25 | 0.965 | 0.5327 |
| FrSBe self total | pwALS | 23 | 0.912 | **0.0453** |
| FrSBe self Apathy | pwALS | 23 | 0.964 | 0.5575 |
| FrSBe self Disinhibition | pwALS | 23 | 0.954 | 0.3469 |
| FrSBe self Executive dysfunction | pwALS | 23 | 0.929 | 0.1042 |
| FrSBe care total | pwALS | 26 | 0.95 | 0.227 |
| FrSBe care Apathy | pwALS | 26 | 0.901 | **0.0167** |
| FrSBe care Disinhibition | pwALS | 26 | 0.925 | 0.0588 |
| FrSBe care Executive dysfunction | pwALS | 26 | 0.95 | 0.236 |
| Age | HC | 74 | 0.952 | **0.007** |
| DAS care total | HC | 68 | 0.926 | **0.0006** |
| DAS care Executive | HC | 68 | 0.845 | **0** |
| DAS care Emotional | HC | 68 | 0.962 | **0.0375** |
| DAS care Initiation | HC | 68 | 0.968 | **0.0806** |
| DAS self total | HC | 74 | 0.905 | **0** |
| DAS self Executive | HC | 74 | 0.891 | **0** |
| DAS self Emotional | HC | 74 | 0.968 | 0.0589 |
| DAS self Initiation | HC | 74 | 0.905 | **0** |
| HADS-D total | HC | 74 | 0.949 | **0.0048** |
| HADS-D Anxiety | HC | 74 | 0.944 | **0.0025** |
| HADS-D Depression | HC | 74 | 0.944 | **0.0025** |
| AES self | HC | 69 | 0.837 | **0** |
| AES care | HC | 62 | 0.867 | **0** |
| FrSBe self total | HC | 71 | 0.947 | **0.0049** |
| FrSBe self Apathy | HC | 71 | 0.925 | **0.0004** |
| FrSBe self Disinhibition | HC | 71 | 0.979 | 0.2748 |
| FrSBe self Executive dysfunction | HC | 71 | 0.921 | **0.0003** |
| FrSBe care total | HC | 59 | 0.964 | 0.0795 |
| FrSBe care Apathy | HC | 59 | 0.943 | **0.0082** |
| FrSBe care Disinhibition | HC | 59 | 0.964 | 0.0761 |
| FrSBe care Executive dysfunction | HC | 59 | 0.965 | 0.0865 |

Supplementary Table 2: Item-subscale correlations

| Subscale | Self-rated |  | Caregiver-rated |  |
| --- | --- | --- | --- | --- |
| Emotional | r.cor | Mean ± sd | r.cor | Mean ± sd |
| Item 3 | .54 | 0.85 ± 0.82 | .60 | 0.91 ± 0.89 |
| Item 5 | .60 | 1.24 ± 0.96 | .59 | 0.81 ± 0.83 |
| Item 7 | .61 | 1.45 ± 0.85 | .70 | 1.31 ± 0.92 |
| Item 9 | .56 | 1.14 ± 0.91 | .48 | 0.97 ± 0.73 |
| Item 12 | .51 | 0.78 ± 0.83 | .70 | 0.78 ± 0.77 |
| Item 15 | .45 | 1.60 ± 0.94 | .54 | 1.71 ± 0.98 |
| Item 20 | .59 | 1.30 ± 0.84 | .31 | 1.53 ± 0.97 |
| Item 24 | .51 | 0.42 ± 0.60 | .60 | 0.49 ± 0.63 |
|  |  |  |  |  |
| Initiation |  |  |  |  |
| Item 2 | .29 | 1.27 ± 0.82 | .49 | 1.12 ± 0.85 |
| Item 4 | .73 | 1.03 ± 0.83 | .58 | 0.99 ± 0.74 |
| Item 8 | .70 | 0.78 ± 0.76 | .64 | 0.79 ± 0.80 |
| Item 13 | .82 | 0.69 ± 0.74 | .65 | 0.56 ± 0.70 |
| Item 14 | .69 | 1.23 ± 0.80 | .73 | 1.10 ± 0.69 |
| Item 16 | .67 | 0.93 ± 0.76 | .61 | 0.78 ± 0.67 |
| Item 18 | .36 | 0.68 ± 0.86 | .43 | 0.53 ± 0.70 |
| Item 22 | .50 | 1.37 ± 0.82 | .30 | 1.40 ± 0.85 |
|  |  |  |  |  |
| Executive |  |  |  |  |
| Item 1 | .67 | 0.54 ± 0.71 | .62 | 0.50 ± 0.61 |
| Item 6 | .64 | 0.27 ± 0.56 | .53 | 0.19 ± 0.43 |
| Item 10 | .76 | 0.61 ± 0.87 | .53 | 0.37 ± 0.62 |
| Item 11 | .80 | 0.65 ± 0.77 | .74 | 0.47 ± 0.68 |
| Item 17 | .35 | 0.73 ± 0.88 | .49 | 0.37 ± 0.67 |
| Item 19 | .60 | 0.60 ± 0.68 | .50 | 0.69 ± 0.83 |
| Item 21 | .75 | 0.66 ± 0.73 | .66 | 0.37 ± 0.62 |
| Item 23 | .67 | 0.92 ± 0.70 | .76 | 0.56 ± 0.68 |

Supplementary Table 3: Median. IQR. and range of self and caregiver subscales in HC and pwALS (corresponding to the Boxplots displayed in Figure 1)

| ***Rater*** | **Group** | **Subscale** | **Median** | **IQR** | **Range** |
| --- | --- | --- | --- | --- | --- |
| *Self* | HC | Executive | 4.0 | 1.8-6 | 0-17 |
| *Self* | pwALS | Executive | 6.5 | 3-11.5 | 0-17 |
| *Self* | HC | Emotional | 9.5 | 6-12 | 2-17 |
| *Self* | pwALS | Emotional | 11.0 | 7.8-12 | 2-17 |
| *Self* | HC | Initiation | 7.0 | 4.8-8.2 | 2-15 |
| *Self* | pwALS | Initiation | 10.5 | 6-14 | 0-23 |
| *Caregiver* | HC | Executive | 3.0 | 0.2-4 | 0-15 |
| *Caregiver* | pwALS | Executive | 5.0 | 2.2-13.8 | 0-19 |
| *Caregiver* | HC | Emotional | 8.0 | 5-10.8 | 1-16 |
| *Caregiver* | pwALS | Emotional | 9.0 | 8-16 | 0-20 |
| *Caregiver* | HC | Initiation | 5.5 | 4.2-8.8 | 1-12 |
| *Caregiver* | pwALS | Initiation | 13.0 | 9.5-17 | 1-22 |

Supplementary Figure 1: Detailed flow-chart of the translational process. During this process, items were reviewed for linguistic clarity and cultural relevance. No substantial cultural adaptations were required as the items were considered applicable in the German context; only minor linguistic refinements were made to improve clarity and natural phrasing (i.e., Item 15 was phrased positively rather than the original DAS version).
